# Associations of domain‐specific physical activities with insomnia symptoms among 0.5 million Chinese adults

**DOI:** 10.1111/jsr.12507

**Published:** 2017-02-23

**Authors:** Bang Zheng, Canqing Yu, Liling Lin, Huaidong Du, Jun Lv, Yu Guo, Zheng Bian, Yiping Chen, Min Yu, Jianguo Li, Junshi Chen, Zhengming Chen, Liming Li, The China Kadoorie Biobank Collaborative Group

**Affiliations:** ^1^ Department of Epidemiology and Biostatistics School of Public Health Peking University Health Science Center Beijing China; ^2^ Clinical Trial Service Unit & Epidemiological Studies Unit (CTSU) Nuffield Department of Population Health University of Oxford Oxford UK; ^3^ Chinese Academy of Medical Sciences Beijing China; ^4^ Zhejiang Center for Disease Control and Prevention Administration Office Hangzhou Zhejiang China; ^5^ Pengzhou Center for Disease Control and Prevention Pengzhou Sichuan China; ^6^ China National Center for Food Safety Risk Assessment Beijing China

**Keywords:** exercise, gender heterogeneity, relationship, sleep complaints

## Abstract

Previous studies have demonstrated the association between physical activity and sleep quality. However, there is little evidence regarding different domains of physical activity. This study aimed to examine the associations between domain‐specific physical activities and insomnia symptoms among Chinese men and women. Data of 452 024 Chinese adults aged 30–79 years from the China Kadoorie Biobank Study were analysed. Insomnia symptoms were assessed with self‐reported difficulties in initiating or maintaining sleep, early morning awakening, daytime dysfunction and any insomnia symptoms. Physical activity assessed by questionnaire consisted of four domains, including occupational, commuting‐related, household and leisure‐time activities. Gender‐specific multiple logistic regression models were employed to estimate independent associations of overall and domain‐specific physical activities with insomnia symptoms. Overall, 12.9% of men and 17.8% of women participants reported having insomnia symptoms. After adjustment for potential confounders, a moderate to high level of overall activity was associated with reduced risks of difficulties in initiating or maintaining sleep and daytime dysfunction in both sexes (odds ratios range: 0.87–0.94, *P *< 0.05). As to each domain of physical activity, similar associations were identified for occupational, household and leisure‐time activities in women but not men (odds ratios range: 0.84–0.94, *P *< 0.05). Commuting‐related activity, however, was associated with increased risks of difficulties in initiating or maintaining sleep and any insomnia symptoms in both sexes (odds ratios range: 1.07–1.17, *P *< 0.05). In conclusion, a moderate to high level of physical activity was associated with lower risks of insomnia symptoms among Chinese adults. However, such associations varied hugely in different domains of physical activity and with gender differences, which could help with better policy‐making and clinical practice.

## Introduction

Insomnia, often known as poor sleep quality of having difficulties in sleep initiation or sleep maintenance (DIMS), accompanied by daytime dysfunction (DDF), is one of the most common sleep disorders throughout the world (Leger *et al*., 2008; Sateia, 2014; Sateia *et al*., 2000). Results from the 2007 China Chronic Disease and Risk Factor Surveillance showed that 35.7% of Chinese residents aged 15–69 years reported poor sleep quality (Yin *et al*., 2011). Existing epidemiological studies have shown that insomnia and related sleep problems have both short‐term and long‐term adverse effects on mental health as well as physical health (Fernandez‐Mendoza and Vgontzas, 2013). Recent evidence from several large prospective studies also associated insomnia with adverse health outcomes, such as all‐cause mortality, cardiovascular diseases and metabolic disorders (Lallukka *et al*., 2016; Sofi *et al*., 2014).

Physical activity has long been examined as a lifestyle factor that could improve sleep quality in the general population as well as in specific patient populations (Driver and Taylor, 2000; Kubitz *et al*., 1996). Moderate regular exercise is generally recommended as part of the sleep hygiene education (Morin *et al*., 1999). Although there existed some doubts and arguments (Trinder *et al*., 1988), a variety of epidemiological and experimental studies have demonstrated that regular exercise could reduce the prevalence of sleep disorders, increase slow‐wave sleep time and decrease sleep latency (Driver and Taylor, 2000).

However, only several previous studies have examined the associations with respect to different domains of physical activity. As participation in occupational, recreational, household or commuting activities may differ considerably in energy cost and physiological impact, it was hypothesized that their effects on sleep and the underlying mechanisms could be different (Morgan, 2003). For instance, recreational activities may contribute to sleep quality through their influence on psychological health and life satisfaction. On the contrary, Costa *et al*. (1988) found that excessive commuting‐related activity could do harm to sleep for working people through restricting sleep time and increasing psychological stress. Therefore, simply focusing on leisure‐time exercise or overall physical activity, as was done in most previous studies, may not be sufficient enough to clarify the relationships between sleep and physical activity. Moreover, there is a lack of evidence in the Chinese population regarding the associations between physical activity and specific insomnia symptoms. Evidence showed that the architecture of physical activity among the Chinese population is entirely different from that in Western countries. Instead of leisure‐time exercise, physical activity of Chinese adults mainly involves occupation and housework (Bauman *et al*., 2008; Du *et al*., 2014; Ng *et al*., 2009).

Therefore, the present study was conducted to clarify the relationships between different domains of physical activity and insomnia symptoms among Chinese adults, as well as to examine potential gender differences in the relationships.

## Materials and methods

### Study population

This study was performed using the survey data from the China Kadoorie Biobank (CKB) Study, a large population‐based study that aims to assess the complex interplay of lifestyle, environmental and genetic factors as determinants of chronic diseases. Details of the CKB study have been previously described (Chen *et al*., 2005; Li *et al*., 2012). Briefly, the CKB study included 512 891 adults (aged 30–79 years) from five urban and five rural areas across China, who had completed a survey during 2004–2008. Subjects were excluded from the current analyses if they had a history of stroke (*n *= 8884), coronary heart disease (*n *= 15 472), chronic obstructive pulmonary disease (COPD; *n *= 37 063) or cancer (*n *= 2577). Those with missing or wrong information on the physical activity or sleep quality metrics (*n *= 894) were also excluded. After exclusions, 452 024 participants (181 749 men and 270 275 women) were included in the analyses. The study was approved by the Ethical Review Committee of the Chinese Center for Disease Control and Prevention (Beijing, China) and the Oxford Tropical Research Ethics Committee, University of Oxford (UK). All participants provided written informed consent.

### Insomnia symptoms and physical activity assessment

The CKB survey included questions pertaining to sleep quality and domain‐specific physical activities. All of the sleep quality metrics were assessed in reference to the past month, except for snoring frequency. Among these metrics, four insomnia symptom categories were constructed according to previous studies (Sherrill *et al*., 1998). Subjects were classified as having DIMS if they reported ‘having trouble falling asleep (sleep onset latency ≥30 min) after going to bed or waking up in the middle of the night at least 3 days a week’; those who reported ‘waking up too early and not be able to get back to sleep at least 3 days a week’ were classified as having problems of early morning awakening (EMA); those reporting ‘having trouble keeping sober‐minded during daytime because of bad sleep at least 3 days a week’ were classified as having DDF; and those reporting one or more of the three mentioned symptoms were classified as having any insomnia symptoms (AIS).

Physical activity in the past year was estimated via a questionnaire adapted from validated questionnaires previously adopted in several Chinese and Western population studies (Matthews *et al*., 2003; Wareham *et al*., 2002), with some further modifications based on a CKB pilot study to achieve cross‐cultural adaptation and equivalence. A detailed description of physical activity questions has been previously reported, along with the methods used to quantify domain‐specific physical activity levels in metabolic equivalents (MET; Du *et al*., 2013). In brief, participants were asked about their usual type and duration of physical activities related to work, commuting, household chores and leisure‐time exercise during the past year. It is to be noted that commuting‐related activity referred to physical activity during the journey between home and working place only, and leisure‐time activity did not include sedentary activities such as watching television and reading. The MET value of a certain type of activity represents the ratio of the energy expended per kilogram of body weight during that activity to that expended while sitting quietly. The MET value of each activity was multiplied by the hours spent per day performing that activity (Ainsworth *et al*., 2011). The overall physical activity level was then calculated by summing the MET‐hours of four domains of physical activities.

In addition, information on several covariate factors was also collected during the survey, including socio‐demographic factors (age, gender, geographical region, education level, marital status, annual household income), body mass index [BMI; weight (kg)/(height (m))^2^], lifestyle factors (smoking status, alcohol and tea consumption, dietary consumption), mental health status and menopause status (only for women), which were examined as potential confounders or effect modifiers in the analyses. As for mental health status, four major depression symptoms (feeling sad or depressed; loss of interest; loss of appetite; and feeling worthless or useless for more than 2 weeks) and one anxiety symptom (feeling continuously worried, tense or anxious that interfered your life for 1 month or longer) were assessed by questionnaire during the survey.

### Statistical analysis

Descriptive analyses were conducted to examine gender differences in physical activity and insomnia symptoms. The mean level of overall physical activity in MET‐hours/day was estimated in the male and female population. For each of the four domain‐specific activities, subjects were divided into two groups: having performed the activity or not. The proportion of subjects having not performed the activity and the mean level of activity in MET‐hours/day were estimated separately.

Multiple logistic regression models were then employed to estimate independent associations between physical activity and insomnia symptoms (DIMS, EMA, DDF, AIS). Each insomnia symptom was analysed in two separate models. In the first model (model 1), overall physical activity level (classified into four groups by quartiles) was evaluated as an independent risk factor, while the second model (model 2) included the four domains of physical activity as independent variables. Occupational, commuting‐related and household activity level were classified into three groups by tertiles, while leisure‐time activity level was classified into no activity group, low and high groups (by the median of those having performed such activities). To be noted, those without work were assigned to a separate category for occupational and commuting‐related activities with no results reported.

All models were gender‐specific and adjusted for age, geographical region (10 study areas), educational level (no formal school, primary school, middle school, and high school or above), marital status (married or else), annual household income (<10 000, 10 000–19 999, 20 000–34 999 and ≥35 000 yuan), BMI, smoking status (never, ex‐regular or occasional, and current smokers), alcohol consumption (never, ex‐regular or occasional, and monthly or weekly), tea consumption (never, occasional, monthly but not weekly, and weekly), dietary intake frequency of fresh fruit, fresh vegetables and dairy products, mental health status (having depression or anxiety symptoms or not), and menopause status (pre‐menopause, peri‐menopause and post‐menopause, only for women). Furthermore, heterogeneity between genders was tested with interaction terms in models including both men and women participants.

Finally, we assessed the robustness of estimates by conducting several sensitivity analyses: stratifying analyses by age groups (dichotomized at age 50 years because the mean age was 50.5 years); restricting analyses to participants without respiratory diseases and rheumatoid arthritis; excluding participants with frequent use of sleep aid medications; additionally adjusting for medication use for hypertension and diabetes. All statistical analyses were conducted using SAS 9.3 (SAS Institute, Cary, NC, USA). Where applicable, a *P*‐value of less than 0.05 was considered statistically significant.

## Results

### Descriptions of insomnia symptoms and physical activity levels

Of the 452 024 subjects included in the analyses, 40.2% were men, the mean age was 50.5 ± 10.4 years, and the mean BMI was 23.7 ± 3.3 kg m^−2^. Overall, 12.9% of male and 17.8% of female participants reported having AIS. The prevalence rates of the three categories of insomnia symptoms (DIMS, EMA, DDF) in women were all relatively higher than that of men (*P *< 0.05). The symptom of DDF was relatively less reported than the other two in both sexes (Table 1).

**Table 1:** Descriptive insomnia and physical activity metrics by gender

Variables	Men (*n *= 181 749)	Women (*n *= 270 275)	Total (*n *= 452 024)
Insomnia symptoms, %			
DIMS	8.7	12.6	11.0
EMA	7.9	11.4	10.0
DDF	1.4	2.6	2.1
AIS	12.9	17.8	15.9
Physical activity, mean ± SD			
Overall level, MET‐hours/day	22.9 ± 15.1	20.9 ± 12.8	21.7 ± 13.8
Occupational activity			
No activity, %	18.3	34.8	28.2
Mean level, MET‐hours/day^a^	21.2 ± 13.8	16.8 ± 12.0	18.8 ± 13.0
Commuting‐related activity			
No activity, %	31.4	47.5	41.0
Mean level, MET‐hours/day^a^	2.3 ± 2.4	2.2 ± 2.4	2.2 ± 2.4
Household activity			
No activity, %	21.0	0.7	8.9
Mean level, MET‐hours/day	2.8 ± 2.8	7.7 ± 3.9	5.7 ± 4.2
Leisure‐time activity			
No activity, %	78.6	80.2	79.6
Mean level, MET‐hours/day	0.8 ± 2.2	0.8 ± 2.2	0.8 ± 2.2

AIS, any insomnia symptoms; DDF, daytime dysfunction; DIMS, difficulties in initiating or maintaining sleep; EMA, early morning awakening; MET, metabolic equivalents.

a The means and SDs were calculated for working people only.

The mean overall physical activity level (in MET‐hours/day) was 22.9 ± 15.1 in men and 20.9 ± 12.8 in women. Occupational activity had the largest contribution to the overall physical activity level, and was higher in men (21.2 ± 13.8) than women employees (16.8 ± 12.0, *P *< 0.05). Leisure‐time exercise was the smallest domain, with 78.6% of men and 80.2% of women reported having no such activities during the past year. There were 21.0% of men having not performed household activity while the proportion was only 0.7% in women, the average household activity level was much higher in women population as well (*P *< 0.05; Table 1).

### Association of overall physical activity level with insomnia symptoms

The results of the multiple logistic regression modelling (model 1) are listed in Table 2. For AIS, moderate level of overall physical activity (Q3 group) was associated with a slightly reduced risk in women [odds ratio (OR): 0.96, 95% confidence interval (CI): 0.93–0.99, *P *< 0.05], while no significant association was found in men. For both sexes, moderate to high levels of overall physical activity (Q3 and/or Q4 groups) were associated with lower risks of DIMS and DDF (ORs range: 0.87–0.94, *P *< 0.05). No gender differences were found for any previous associations (*P *> 0.05).

**Table 2:** Associations between overall physical activity and insomnia symptoms by gender

Overall physical activity level	DIMS		EMA		DDF		AIS	
	*N* (%)	OR (95% CI)	*N* (%)	OR (95% CI)	*N* (%)	OR (95% CI)	*N* (%)	OR (95% CI)
Men								
Q1	4557 (9.9)	1	4225 (9.2)	1	686 (1.5)	1	6623 (14.4)	1
Q2	3318 (8.8)	0.99 (0.95–1.04)	3141 (8.3)	1.07 (1.02–1.13)	529 (1.4)	1.02 (0.90–1.15)	4982 (13.2)	1.04 (0.99–1.08)
Q3	3503 (7.9)	0.94 (0.89–0.98)	3223 (7.3)	1.03 (0.97–1.08)	554 (1.3)	0.87 (0.77–0.98)	5312 (12.1)	0.99 (0.95–1.03)
Q4	4400 (8.2)	0.96 (0.91–1.01)	3792 (7.0)	1.03 (0.98–1.09)	677 (1.3)	0.87 (0.77–0.98)	6527 (12.1)	1.01 (0.97–1.06)
Women								
Q1	9759 (14.6)	1	8498 (12.7)	1	1832 (2.7)	1	12 999 (19.4)	1
Q2	10 170 (13.5)	0.99 (0.96–1.02)	9024 (12.0)	1.03 (1.00–1.07)	2025 (2.7)	0.99 (0.93–1.06)	13 952 (18.6)	1.02 (0.98–1.05)
Q3	7881 (11.4)	0.88 (0.85–0.92)	7608 (11.0)	1.00 (0.97–1.04)	1958 (2.8)	0.96 (0.89–1.03)	11 846 (17.2)	0.96 (0.93–0.99)
Q4	6146 (10.4)	0.92 (0.88–0.95)	5747 (9.7)	1.01 (0.97–1.06)	1277 (2.2)	0.88 (0.81–0.96)	9427 (15.9)	1.02 (0.98–1.05)
*P* for gender heterogeneity	0.912		0.529		0.146		0.140	

AIS, any insomnia symptoms; CI, confidence interval; DDF, daytime dysfunction; DIMS, difficulties in initiating or maintaining sleep; EMA, early morning awakening; OR, odds ratio.

*Note*: models were adjusted for age, region, educational level, marital status, annual household income, BMI, smoking status, alcohol consumption, tea consumption, dietary intake frequency of fresh fruit, vegetables and dairy products, mental health status, and menopause status (only for women).

### Associations of domain‐specific physical activities with insomnia symptoms

To clarify the independent associations of domain‐specific physical activities with insomnia symptoms, further modelling (model 2) was performed, with the results presented in Table 3. For AIS, moderate or high levels of occupational, household and leisure‐time physical activities were associated with lower risks in women (ORs range: 0.89–0.94, *P *< 0.05). While in both sexes, moderate and high levels of commuting‐related activity (T2 and T3 groups) were associated with higher risks of AIS (ORs range: 1.07–1.14, *P *< 0.05).

**Table 3:** Associations of domain‐specific physical activities with insomnia symptoms by gender (OR, 95% CI)

Physical activity categories	DIMS		EMA		DDF		AIS	
	Men	Women	Men	Women	Men	Women	Men	Women
Occupational activity^a^								
T1	1	1	1	1	1	1	1	1
T2	0.99 (0.94–1.04)	0.89 (0.85–0.92)	0.96 (0.91–1.02)	0.94 (0.90–0.98)	1.03 (0.91–1.17)	0.94 (0.87–1.02)	0.98 (0.93–1.02)	0.92 (0.89–0.95)
T3	0.96 (0.91–1.01)	0.94 (0.90–0.99)	0.96 (0.91–1.01)	0.99 (0.95–1.04)	0.93 (0.82–1.05)	0.89 (0.81–0.97)	0.96 (0.92–1.01)	0.99 (0.95–1.03)
*P* for gender heterogeneity	< 0.001		< 0.001		0.637		< 0.001	
Commuting‐related activity^a^								
T1	1	1	1	1	1	1	1	1
T2	1.09 (1.04–1.14)	1.17 (1.12–1.21)	1.04 (0.99–1.10)	1.07 (1.03–1.11)	0.90 (0.79–1.01)	0.96 (0.88–1.03)	1.07 (1.03–1.11)	1.14 (1.10–1.18)
T3	1.08 (1.03–1.13)	1.15 (1.10–1.20)	1.01 (0.96–1.07)	1.01 (0.97–1.06)	1.02 (0.91–1.15)	1.06 (0.98–1.15)	1.07 (1.03–1.12)	1.11 (1.07–1.15)
*P* for gender heterogeneity	< 0.001		0.032		0.495		< 0.001	
Household activity								
T1	1	1	1	1	1	1	1	1
T2	0.96 (0.92–0.99)	0.87 (0.82–0.93)	1.08 (1.04–1.12)	0.98 (0.92–1.05)	0.99 (0.91–1.09)	0.89 (0.78–1.01)	0.99 (0.97–1.03)	0.92 (0.88–0.97)
T3	0.98 (0.91–1.04)	0.89 (0.83–0.94)	1.10 (1.03–1.18)	1.02 (0.95–1.09)	0.96 (0.82–1.13)	0.88 (0.77–1.00)	0.98 (0.93–1.04)	0.94 (0.90–1.00)
*P* for gender heterogeneity	0.052		0.034		0.374		0.049	
Leisure‐time activity^b^								
No	1	1	1	1	1	1	1	1
Low	1.04 (0.98–1.10)	1.03 (0.98–1.08)	1.15 (1.08–1.22)	1.01 (0.97–1.06)	0.95 (0.83–1.10)	1.00 (0.91–1.09)	1.09 (1.04–1.15)	1.02 (0.98–1.07)
High	0.95 (0.89–1.02)	0.92 (0.88–0.96)	0.98 (0.92–1.05)	0.89 (0.85–0.93)	0.95 (0.81–1.11)	0.84 (0.76–0.92)	0.96 (0.91–1.01)	0.89 (0.86–0.93)
*P* for gender heterogeneity	0.172		0.020		0.316		0.005	

AIS, any insomnia symptoms; DDF, daytime dysfunction; DIMS, difficulties in initiating or maintaining sleep; EMA, early morning awakening.

Note: models were adjusted for age, region, educational level, marital status, annual household income, BMI, smoking status, alcohol consumption, tea consumption, dietary intake frequency of fresh fruit, vegetables and dairy products, mental health status, and menopause status (only for women).

a Those without work were assigned to a separate category for occupational activity and commuting‐related activity, whose model results were not reported in the table.

b Leisure‐time activity level was classified into no activity group, low and high groups (by the median of those having performed such activities).

Associations of moderate or high levels of occupational activity with lower risks of DIMS, EMA and DDF were identified only in women (ORs range: 0.89–0.94, *P *< 0.05). Moderate and high levels of commuting‐related activity, however, were associated with increased risks of DIMS in both sexes (ORs range: 1.08–1.17, *P *< 0.05). Associations of moderate or high levels of household activity with lower risks of DIMS were identified in both sexes (ORs range: 0.87–0.96, *P *< 0.05), while higher risks of EMA were found in men with moderate and high levels of household activity (ORs range: 1.08–1.10, *P *< 0.05). A high level of leisure‐time activity was associated with lower risks of DIMS, EMA and DDF in women (ORs range: 0.84–0.92, *P *< 0.05). Except for DDF, significant gender differences were detected in most of the associations between domain‐specific activities and insomnia symptoms (*P *< 0.05).

In the sensitivity analyses, the associations of domain‐specific physical activities with insomnia symptoms were consistently observed among the young and the old‐aged populations (<50 or ≥50 years). The estimates remained robust after excluding participants with respiratory diseases and rheumatoid arthritis, or excluding participants with frequent use of sleep aid medications, or additionally adjusting for medication use for hypertension and diabetes.

## Discussion

In this first ever large and detailed epidemiological study of insomnia symptoms in relation to self‐reported overall and domain‐specific physical activities, we confirmed that to a certain extent, moderate to high levels of physical activity were associated with lower risks of insomnia symptoms among Chinese adults. We also identified varied associations between domain‐specific physical activities and sleep, especially the association of commuting‐related activity with higher risks of insomnia symptoms in both sexes.

Our results of the association between overall physical activity and insomnia symptoms are consistent with previous literature (Driver and Taylor, 2000; Kubitz *et al*., 1996; Sherrill *et al*., 1998). For instance, a population‐based study (Sherrill *et al*., 1998) showed that both men and women with regular activity had a 29% reduced risk of disorder in maintaining sleep and a 38% reduced risk of any sleep disorder.

We identified gender differences in the relationships between domain‐specific physical activities and insomnia symptoms, which is partly in line with a meta‐analysis (Kubitz *et al*., 1996) that demonstrated a larger positive impact of exercise on sleep in women than in men. In this study, a moderate or high level of occupational, household and leisure‐time physical activities was associated with lower risks of insomnia symptoms in women, while inverse associations of household and leisure‐time activities with EMA were identified in men. This could be explained by gender heterogeneity and different construct of physical activity domains between men and women (Bauman *et al*., 2008; Du *et al*., 2014; Ng *et al*., 2009). It is also plausible that different symptoms of insomnia reflect different dimensions of sleep architecture and status; thus, their relationships with physical activities might vary under certain physiological mechanisms. In particular, EMA reflects rhythmic disruption during the circadian cycle, and there was evidence that skeletal muscle and tissue damage caused by long, strenuous physical activity might raise the level of inflammatory cytokines that are probably involved in sleep regulation (Driver and Taylor, 2000; Krueger *et al*., 2011). Moreover, the symptom of EMA is more prevalent among patients with COPD or rheumatoid arthritis (Gibbs and Ray, 2013; Stephenson *et al*., 2015), implying possible confounding effects; whereas we have already excluded patients with COPD from analysis, and the association remained in the sensitive analysis after further exclusion of patients with other chronic conditions.

There are several proposed biological mechanisms relating physical activity to improved sleep quality. First, physical activity could benefit sleep through increasing body and central nervous system temperature, which is related to the hypothesized sleep function of thermoregulation, and the following body‐cooling process thus induces sleep onset and promotes sleep quality (Driver and Taylor, 2000; Horne and Staff, 1983). Second, it was hypothesized that sleep also serves the function of energy conservation and body restoration, on which physical activity could have a major impact (Berger and Phillips, 1988). Moreover, outdoor activities provide sufficient bright light exposure, which might promote sleep via circadian phase‐shifting effects and antidepressant effects (Youngstedt, 1997). Other possible mechanisms such as anxiety reduction and brain serotonin metabolism regulation were also suggested (Sherrill *et al*., 1998). To be noted, the timing of physical activity, which is related to the process of body temperature regulation or bright light exposure, could also influence the effects of aforementioned mechanisms. Overall, the underlying physiological mechanisms are not fully understood and still need further research.

As for the associations of excessive commuting‐related activity with higher risks of insomnia symptoms in both sexes, our results are concordant with previous literature. A few epidemiological studies have suggested that daily commuting is an extra stress factor for working people through restricting free‐time and reducing sleep time (Costa *et al*., 1988). Longer trips were also associated with increased sleep problems and psychosomatic complaints, possibly due to elevated psychological stress (Costa *et al*., 1988; Hansson *et al*., 2011). In line with these studies, our study identified stronger associations in the female population. On the other hand, in contrast to the positive associations of other domains of physical activity, the energy cost and physiological impact of commuting‐related activity may be different. Moreover, the means of transportation and the exposure to traffic‐related noise and air pollution could also matter (Fang *et al*., 2015; Hansson *et al*., 2011; Kim *et al*., 2012), which still needs further research.

Although the sample size is quite large and there are plenty of established and potential confounding factors controlled, this study still has several limitations. First, the measurement of insomnia is not entirely corresponding to clinical diagnosis criteria (e.g. the International Classification of Sleep Disorders, ICSD‐3), though specific symptoms of insomnia were well collected. Actually, the definitions of insomnia symptoms in this study were quite strict with the minimum frequency of three times a week during the past month. Second, the reliability and validity of the physical activity questionnaire have not been fully tested yet. Therefore, our findings should be interpreted with caution due to potential information bias. Nonetheless, about 5% of participants from the CKB study were randomly selected to resurvey in 2008 (Chen *et al*., 2011), and the intraclass correlation coefficients for the overall and domain‐specific physical activity levels (in MET‐hours/day) indicated good reliability of survey data. Moreover, the potential measurement error of physical activity level in this study was unlikely to depend on the insomnia symptoms, thus could only bias the results towards null hypotheses. Another limitation is that the study did not collect information about the timing of physical activity, which matters because several studies showed inconsistent results towards possible sleep disruption following acute evening exercise (Buman *et al*., 2014; Oda and Shirakawa, 2014). Finally, the causal relationships could hardly be established in this cross‐sectional analysis, with several previous studies suggesting that sleep quality could reversely influence the motivation of physical activity (Dzierzewski *et al*., 2014; Lambiase *et al*., 2013). However, the physical activity levels that participants reported in this study were in reference to the past year, while the sleep symptoms were asked for the past month, which makes the results of this study more convincing.

In conclusion, moderate to high levels of physical activity were associated with lower risks of insomnia symptoms among Chinese adults. Nevertheless, such associations varied hugely in different domains of physical activity and with significant gender differences. Further research on this issue could help develop and modify the physical activity intervention strategies for promoting sleep quality in the general population. Clinicians should also bear this in mind when providing behavioural therapies or lifestyle advice for patients with sleep disorders.

## Authors’ Contributions

B. Z., C. Y. and L. M. L. designed the study. B. Z. and C. Y. conducted the analyses and drafted the first version of the manuscript. L. L. L. helped with the analyses and data review. H. D., J. L., Y. G., Z. B., Y. C., M. Y., J. G. L., J. C. and Z. C. critically reviewed and revised the manuscript. All authors approved the final version to be published.

## Conflicts of Interest

The authors declare that they have no conflict of interest.
